# Prioritize carbon pricing over fossil-fuel subsidy reform

**DOI:** 10.1016/j.isci.2023.108584

**Published:** 2023-11-25

**Authors:** Jeroen van den Bergh, Cees van Beers, Lewis C. King

**Affiliations:** 1 Institute of Environmental Science and Technology, Universitat Autònoma de Barcelona, UAB campus, 08193 Bellaterra, Spain; 2 ICREA, Passeig de Lluís Companys 23, 08010 Barcelona, Spain; 3 School of Business and Economics & Institute of Environmental Studies, Vrije Universiteit, De Boelelaan 1105, Amsterdam 1081 HV, the Netherlands; 4 Faculty of Technology, Policy and Management, Economics of Technology and Innovation, Delft University of Technology, Jaffalaan 5, 2628 BX Delft, the Netherlands

**Keywords:** Global change, Environmental policy

## Abstract

While many climate activist groups enthusiastically advocate for the removal of fossil-fuel subsidies, we argue that this overstates both the climate effectiveness and political feasibility of such a strategy. Through synthesizing information from various global studies, we show that subsidies contribute to a relatively small portion of climate change and local externality problems, likely accounting for around 1%. We further argue that reform of fossil-fuel subsidies is hampered by various political and social factors, more so than the diffusion of carbon pricing. Based on these results, we argue that the far greater problem of unpriced externalities warrants a redirection or expansion of the enthusiasm for subsidy reform toward carbon pricing. This makes sense also as subsidy reform and carbon pricing essentially represent two sides of the same coin since both contribute to climate mitigation by raising fossil-fuel prices.

## Graphical abstract

**Figure:**
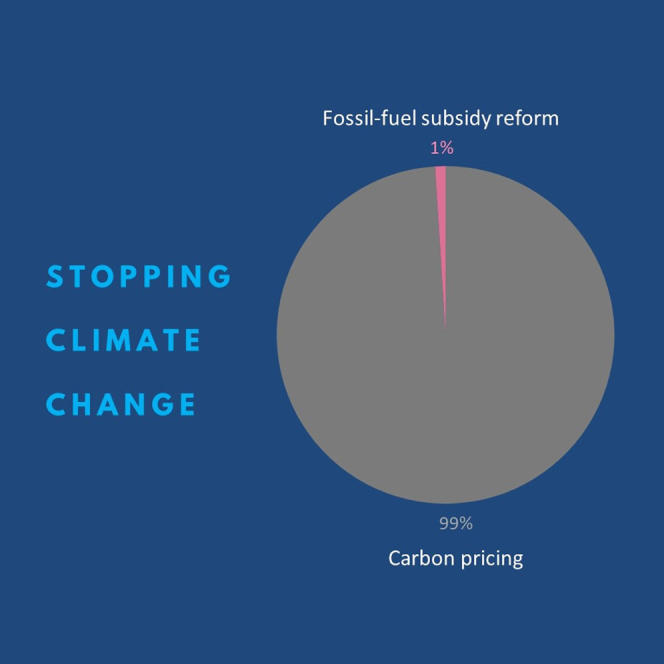

## Introduction

Climate activists in various countries rallied against fossil fuel use earlier this year, with roadblock protests organized by Extinction Rebellion in the Netherlands specifically targeting fossil-fuel subsidies.^1^ We question whether these protests are aimed at the most effective target.

There is considerable uncertainty surrounding the effectiveness of removing fossil-fuel subsidies as a strategy for climate change mitigation. This uncertainty arises from conflicting interpretations of the subsidies themselves as well as the associated ambiguity regarding their actual magnitude. Consequently, the exact impact of removing these subsidies on CO_2_ emissions remains unclear. The question at hand is how much these uncertainties matter to policy recommendations.

We argue that calls for phasing out subsidies on fossil fuels overstate the potential benefits of this strategy, both in terms of its effectiveness in cutting emissions and its political feasibility. By illustrating the relative importance of unpriced climate change externalities versus fossil-fuel subsidies, we propose that a similar – or even greater – level of enthusiasm among activists should be directed toward the widespread implementation of carbon pricing, which is notably absent in the demands of climate activist group like Extinction Rebellion. We stress that we are not against the removal of fossil-fuel subsidies – whenever feasible this should be done. Our point is, however, that its contribution to a climate solution is small compared to that of carbon pricing. The latter therefore merits serious attention from activists.

## Climate effectiveness overstated

The growing body of literature on the reform of fossil-fuel subsidies has presented a wide range of estimates regarding its potential impact on reducing global emissions, from as low as 1% to as high as 21%.^2^^,^^3^^,^^4^^,^^5^^,^^6^^,^^7^ The IPCC suggests a range of 1–4% for CO_2_ and up to 10% for GHGs by 2030 8. Employing five distinct models to guarantee robust results, Jewell et al. (2018)^4^ concluded that the removal of fossil-fuel subsidies would lead to a reduction in global CO_2_ emissions ranging from 1 to 7% by 2030, regardless of whether oil prices are low or high. This falls far short of the 40% reduction required by 2030 to achieve the Paris Agreement target of no more than 1.5°C warming. Moreover, the authors note that subsidy removal in developing and emerging economies that are not major oil and gas exporters would have comparatively minimal effects.

According to Parry (2018),^9^ a key reason for Jewell et al.’s modest estimates is the decline in global subsidies over time, from US$ 570 billion in 2013 to US$ 330 billion in 2015. Another contributing factor may be the differing subsidy definitions and demarcations across studies. For instance, Erickson et al. (2020)^10^ argue that the approach by Jewell et al. (2018)^4^ does not adequately account for investment dynamics and the social-political effects of subsidy removal, likely leading to an underestimation of its potential impact.

Regardless, some of these estimates can still be considered slightly optimistic because they overlook carbon leakage.^11^^,^^12^ The idea here is that a localized phase-out of fossil-fuel subsidies would increase domestic energy prices, reducing energy demand in the reforming countries. This would, in turn, reduce global energy prices, stimulating energy demand and associated emissions (carbon leakage) in other countries. Other studies have also suggested that coal will then remain an important source of energy in the developing world and may even increase its share of the energy supply in some regions.^13^ In addition, phasing out fossil-fuel subsidies has an upper bound in terms of mitigation effects while carbon pricing is flexible to achieve any desired mitigation level. In fact, Schwanitz et al.^14^ found that the removal of fossil-fuel subsidies will only have a small long-term impact on a global transition to low-carbon energy unless complemented by stringent regulation or carbon pricing.

## Confusion about subsidies versus unpriced externalities

The overestimation of the impact subsidy reform would have on emissions reductions is even more significant than previous studies suggest due to two factors: the distinction between genuine versus broad interpretations of fossil-fuel subsidies; and the use of a relatively low value of the social cost of carbon.

Building on earlier IMF studies,^2^^,^^15^ Coady et al. (2017) 3 define a broad notion of energy subsidies as: “what we term ‘post-tax subsidies’ – [arising] when consumer prices are below supply costs, plus a ‘Pigouvian’ tax to reflect environmental damages and general consumer taxes.” In other words, they lump together genuine subsidies and unpriced externalities (what they also call “implicit subsidies”) to obtain a measure of total subsidies. Based on this definition, the authors found that these subsidies amounted to over US$ 5.3 trillion in 2015, whereas pre-tax subsidies were estimated to be only US$ 333 billion in 2013. Pre-tax subsidies, therefore, amounted to a mere 6.3% of total subsidies.

This broad notion of energy subsidies is confusing as it lumps together actual energy subsidies and uncharged external costs (Monkelbaan and Steenblik, 2021, footnote 5).^16^ This confounds policy advice: whereas genuine subsidies may require reform, unpriced externalities necessitate some form of pricing in the form of taxation or cap-and-trade. Grouping the two together in a broad notion of subsidies obscures policy needs while incorrectly suggesting that reform is sufficient, when in fact addressing the substantially larger part requires the implementation of carbon pricing.

However, despite the widespread push to remove fossil-fuel subsidies, there has been less enthusiasm for implementing carbon pricing as an effective alternative, that is, with a sufficiently high price reflecting climate externalities. The reason for this may be that subsidies by governments to energy industries (including “Big Oil”) tend to create more animosity than unpriced externalities caused by and falling upon all of us. Nevertheless, the composition of the IMF estimate is a far less convincing motivation for removing fossil-fuel subsidies than for pricing climate externalities.

Adding to this concern is that previous studies on fossil-fuel subsidies have relied on conservative estimates of climate externalities. Relevant studies have used social costs of carbon (SCC) with values ranging from US$ 25 to US$ 60 per ton of carbon dioxide^2^^,^^3^^,^18. In contrast, more recent studies, including reviews and expert opinion surveys, suggest a considerably higher range of values, reaching up to US$ 310 per ton of carbon dioxide.^18^^,^^19^ Accounting for all main tipping points in the climate system would further increase estimates.^20^ Higher SCCs raise the overall externality costs, consequently reducing the relative importance of subsidy costs. Table 1 illustrates this through adapted estimates by the IMF, Jewell et al., and the IPCC. The relative importance of explicit fossil-fuel subsidies comes out as much lower, going in the direction of 1% of the combined impact of subsidies and non-priced externalities. Climate activists often fail to recognize this relatively small contribution of genuine fossil-fuel subsidies to the problem.

**Table 1:** Illustrating the low importance of explicit fossil-fuel subsidies versus unpriced externalities for a higher social cost of carbon (SCC)

Study	Original estimates of explicit subsidy as a percentage of total subsidy	Adapted estimates for higher SCC (US$ 310)
IMF (Coady et al., 2017; Parry et al., 2021)^3^^,^^17^	6.8 and 8%	2.7 and 3.6% (mean value 3.15%)
Jewell et al. (2018),^4^ based on five models	1–7%	0.4–3.2% (mean value 1.8%)
IPCC (2022),^8^ based on a literature review	1–4%	0.4–1.8% (mean value 1.1%)

We calculated the adapted values for IMF, based on an SCC of US$ 310 instead of 40 (year 2014) and 60 (2020), respectively. Here the SCC of US$ 40 reflects the average of the two values mentioned in Coady et al. (2017)^3^: “varying between $37.7 per ton of CO_2_ in 2011 to $42.3 in 2015”. In the two mentioned years, the values of the total subsidy are 4.9 and 5.9, of the explicit subsidy 0.333 and 0.472, and of the unpriced global warming externality 1.078 and 1.711 (in addition they account for local externalities of pollution, accidents and congestion), respectively (all figures are in trillion US$ of the respective year). The adapted externality values are 8.355 (factor 310/40 higher) and 8.840 (310/60 higher), respectively. The resulting lower percentages obtained (third column), 2.7 and 3.6%, come down to fractions 0.40 and 0.45 of the original percentages (second column); we apply these fractions, respectively, to the lower and higher ends of the value ranges for Jewell et al.^4^ and the IPCC^8^ in the third and fourth rows. This approximation is motivated by the fact that these ranges are based on multiple models (Jewell et al.)^4^ and many primary studies (IPCC),^8^ complicating a “disaggregate adaptation” procedure.

## Political barriers against fossil-fuel subsidy reform

There are several reasons why G20 countries have been unable to eliminate fossil-fuel subsidies despite making repeated promises:

1. Governments have the incentive to provide fossil-fuel subsidies to consumers for political reasons. In non- and semi-democratic countries, this takes the form of targeted government spending on fossil-fuel subsidies to appease citizens or “buy votes”. This is clearly indicated by generally low pump prices of gasoline in such countries.^21^ Studies for democratic countries have shown that subsidies correlate negatively with the tenure of democratic leaders,^22^^,^^23^^,^^24^ and there is a limited impact of political leaders on subsidy reform.^25^ Fossil-fuel subsidies are even greater under centralized governments, especially in oil- and gas-producing regions like Russia, Latin America, the Middle East, and North Africa. These findings suggest that drastic policy reform of fossil-fuel subsidies can be challenging and may require regime change in certain countries, involving a switch from non-democratic to democratic political systems.
2. Fossil-fuel subsidies to consumers are especially common in developing countries as an instrument to support low-income energy-poor rural households. Removing them would require complementary policies to address inequities. Although these subsidies are often viewed as a means of fairly sharing the benefits of local energy resources among citizens, many of them disproportionally benefit richer households.^26^^,^^27^^,^^28^ These include politically influential people who may object to reform. The crucial point here is that many households (and hence many voters) will be affected by the removal of subsidies, making it an unattractive and risky political strategy for incumbent politicians.
3. Fossil-fuel subsidies for producers are also prevalent in developed countries. These are often hidden due to their indirect or quasi-fiscal character – an example is the deduction of drilling costs from federal income taxes in the USA to ensure sufficient domestic energy supply.^29^ Not only does the hidden nature of these subsidies contribute to uncertainty about their magnitude and the associated emissions impact but it also affects the perceptions and actions of voters, lobbyists, and politicians. Moreover, producer subsidies drive investments in capital goods, which strengthens sunk costs and lock-in of fossil-fuel production and consumption, in turn, reinforcing resistance against reform.^10^
4. Supply chains matter as well. A high concentration of producers upstream in supply chains makes it easy to organize lobbying power to obtain subsidies. And the further downstream an activity, the less powerful interest groups lobbying for subsidy support are. One reason why consumers have difficulty organizing to reveal their subsidy preferences is that they are numerous and have dispersed interests.

One might argue that some of the feasibility challenges and factors also apply to carbon pricing. Not denying that efforts to implement carbon pricing have seen successes and failures, it is important to understand that all effective and stringent climate policy struggle to get public and political support. It is not evident that carbon pricing meets more resistance,^30^ even though this is sometimes suggested – but we interpret this as the consequence of carbon pricing having received more attention in academic and political discussions because it is widely considered the best option to systemically control carbon emissions. A recent study undertook a public survey in five countries – Ecuador, Egypt, Mexico, Indonesia and India – all of which have substantial consumption- and production-based fossil-fuel subsidies.^31^ It concludes “… we should expect real-life suggestions for subsidy removal to be met with similar public opposition or acceptability as we have seen for other carbon-pricing measures”. A broad in-depth review by Rabe (2018)^32^ provides a balanced account of the diverse experiences and concludes that carbon pricing has many opportunities to be feasible and durable. When comparing the feasibility of fossil-fuel subsidy reform and carbon pricing it is also good to consider that the endowment effect and loss aversion play a role.^33^ This suggests that the removal of FFSs may often be harder to achieve than the implementation of carbon pricing.

In addition, support for fossil fuels is dominated by national processes, while support for carbon pricing has a strong international dimension. The EU ETS covering 30 countries is one indication of this. A study by Skovgaard et al. (2019)^34^ concludes that the reasons for adopting carbon pricing have shifted over time from domestic to international factors. In implementing climate policy, countries worry about their competitiveness and hence which policies other countries have implemented. An advantage of carbon pricing is that it allows easy comparison and harmonization of policies, which in turn can promote its diffusion worldwide.^35^

It is further worthwhile noting that carbon pricing can in fact already count on considerable political feasibility as supported by numerous studies and facts. For example, it has already been applied in 47 national and 36 sub-national jurisdictions.^36^ The EU-ETS is widely considered the most successful carbon pricing system, harmonizing policy across 30 countries and maintaining a relatively high value of around € 80 tCO_2_^−1^ over the past year (and about € 100 tCO_2_^−1^ at the moment of writing this). Hence, the introduction or extension of carbon pricing is not only an effective way to reduce greenhouse gas emissions but arguably politically easier to achieve.

Finally, by selecting the cheapest emission reduction options, carbon pricing works out best for the economy in terms of income, employment, and welfare compared to other instruments.^37^ This can also serve as a factor to garner policy support *ex ante* or continue and increase it ex-post when policy impacts are visible.

## Implications for climate action

To contribute toward achieving the goals of the Paris Agreement, fossil-fuel subsidy reform would require swift action. If one desires to invest time in effectively phasing out fossil-fuel subsidies, a shift in focus from consumer to producer subsidies is recommendable (see also Erickson et al.^10^). Since the latter type is levied early in the value chain, it can have a ripple effect throughout the supply chain, ultimately reducing prices for consumers as well. Producer subsidies, therefore, have a greater impact on total emissions over a product’s life cycle than consumer subsidies. Although focusing on producer subsidies would theoretically be the better strategy, their hidden nature and the effectively organized political resistance against reform cast doubt on a quick and successful outcome. Nevertheless, as we have plausibly argued here, this should be of little concern since genuine fossil-fuel subsidy reform has limited effectiveness for cutting emissions anyway. This does not deny that subsidy removal should be attempted whenever feasible. As we have argued, though, social-political barriers against it may be stronger than is often realized.

In view of this, activists may want to reconsider their strategy by recognizing that the primary cause of excessive carbon emissions – unpriced externalities – is much more important than subsidy reform. Carbon pricing to correct these externalities will achieve considerably more emissions reduction than subsidy reform, particularly in developed countries. This is illustrated by both multi-country studies of large individual countries.^38^ For instance, in the context of China, Mo et al. (2021)^39^ found that even a low carbon price of 50 CNY tCO_2_^−1^ growing at 4% annually would substantially reduce the financial sustainability of coal power. Our illustrative calculations based on global studies in Section Climate effectiveness overstated suggest that carbon pricing is very likely to account for about 99% of the solution. The focus on carbon pricing is further justified due to its already successful implementation in many national and sub-national jurisdictions as well as its potential for easy harmonization across countries. Moreover, a growing body of empirical studies has demonstrated the effectiveness of carbon pricing, with a high emissions-reduction elasticity observed even for relatively low past prices.^40^ A final argument is that carbon pricing achieves much emissions reduction as it directly focuses on the more pollutive activities, sectors, and goods. Altogether, we claim that it is a more effective strategy to achieve the 2030 Paris targets than reforming fossil-fuel subsidies.

Unfortunately, many climate activists are still disinterested or even skeptical about carbon pricing despite it essentially having the same type of effect as subsidy reform. Indeed, they are essentially two sides of the same coin: both contribute to climate mitigation by increasing the prices of fossil fuels. Possibly, climate activists prefer the idea of fossil-fuel subsidy reform because they conceptually view it as a more direct way to hold the “bad guys”, such as “Big Oil”, morally accountable for contributing to climate change.^41^ They appear less aware, however, that both subsidy reform and carbon pricing would in reality have the same effect of increasing the prices of carbon-intensive goods and services for all producers and consumers. In fact, fossil-fuel subsidies can be regarded as “negative carbon prices”.

Based on the arguments and evidence collected here, carbon pricing deserves equal – if not greater – enthusiasm among activists than subsidy reform for tackling climate change. Clearer communication on this matter from both experts and activists could increase public support for carbon pricing, enhancing the political feasibility of its widespread and stringent implementation.
